# Outcomes in Children With Enterovirus Meningitis in London, England: A Retrospective Multicenter Cohort Study, 2013–2023

**DOI:** 10.1093/ofid/ofag112

**Published:** 2026-03-09

**Authors:** Jonathan Broad, Luca Zombori, Blanca Bravo Queipo-de-Llano, Alasdair Bamford, Tim Best, Jonathan Cohen, Simon B Drysdale, Abirami Manian, Neesha Soni, Elizabeth Whittaker, Seilesh Kadambari

**Affiliations:** Department of Paediatric Infectious Diseases, Great Ormond Street Hospital for Children NHS Foundation Trust, London, UK; Paediatric Intensive Care, Department of Heart and Lung, Great Ormond Street Hospital for Children, London, UK; Department of Paediatric Infectious Diseases, Imperial College Healthcare NHS Trust, London, UK; Institute for Health Research of La Paz University Hospital (IdiPAZ), Networked Biomedical Research Centre for Infectious Diseases (CIBERINFEC), Carlos III Health Institute, Autonomous University of Madrid (UAM), Madrid, Spain; Department of Paediatric Infectious Diseases, Great Ormond Street Hospital for Children NHS Foundation Trust, London, UK; Infection, Immunity and Inflammation, University College London Great Ormond Street Institute of Child Health, London, UK; Department of Microbiology, Great Ormond Street Hospital for Children, London, UK; Paediatric Infectious Diseases and Immunology, Evelina London Children's Hospital, London, UK; Department of Paediatrics, St George's University Hospitals NHS Foundation Trust, London, UK; Centre for Neonatal and Paediatric Infection, St George's, University of London, London, UK; Paediatric Infectious Diseases and Immunology, Evelina London Children's Hospital, London, UK; Department of Paediatrics, St George's University Hospitals NHS Foundation Trust, London, UK; Department of Paediatric Infectious Diseases, Imperial College Healthcare NHS Trust, London, UK; Department of Paediatric Infectious Diseases, Great Ormond Street Hospital for Children NHS Foundation Trust, London, UK; Infection, Immunity and Inflammation, University College London Great Ormond Street Institute of Child Health, London, UK

**Keywords:** enterovirus, meningitis, neurodevelopment, pediatrics

## Abstract

Nonpolio enteroviruses are the most common cause of meningitis in children. We conducted a retrospective case series of long-term outcomes in 243 children between 1 January 2013 and 31 December 2023 across 4 tertiary centers in London. Adverse outcomes were associated with the absence of fever at presentation (odds ratio [OR], 4.65; 95% CI, 1.03–20.83), the presence of seizures (OR, 7.40; 95% CI, 1.05–51.96), and the presence of comorbidities at baseline (OR, 5.27; 95% CI, 1.18–23.47). Full recovery was seen in 153 of 160 (95.6%) children who were <3 months of age. These data may help clinicians to counsel parents and policy makers on streamlining care pathways following hospital discharge.

Nonpolio enteroviruses (EVs) are the most common cause of pediatric viral meningitis [1]. In the United Kingdom, in the era of conjugate vaccines, EVs account for more cases of childhood meningitis than all bacterial causes combined [2]. A British Paediatric Surveillance Unit study showed that 11% of infants <90 days of age with EV meningitis required high dependency or intensive care [3].

Long-term outcomes of children with EV meningitis remain poorly understood. A systematic review highlighted only 4 observational cohort studies globally in the last 3 decades that include data on neurodevelopmental sequalae in childhood meningitis caused by non-EV71 infection [4]. The review showed reassuring outcomes but noted limitations, such as small cohorts and the lack of robust follow-up data.

Defining the long-term outcomes of childhood EV meningitis is crucial to guide clinical care (ie, provide prognostic information to children and their families), inform health care provision (ie, inform clinical care pathways beyond hospital discharge), and direct research priorities (ie, treatment and prevention trials). This study examines the long-term outcomes in children with EV meningitis in London, United Kingdom, over a 10-year period.

## METHODS

Cases of EV meningitis were retrospectively identified by interrogating local National Health Service (NHS) virology databases as confirmed by polymerase chain reaction at 4 large pediatric units in London from 1 January 2013 to 31 December 2023. A case of EV meningitis was defined as detection of EV in cerebrospinal fluid (CSF) and may incorporate children with meningitis and/or encephalitis. Cases were cross-checked with hospital discharge coding data. Clinical samples were tested at the corresponding laboratory with polymerase chain reaction assays according to local protocols validated for clinical use. This included in-house and commercial assays. Notable contextual factors that shaped testing practice during the study period include national guidance recommending lumbar puncture in febrile infants <3 months of age and expansion of molecular multiplex diagnostics within NHS laboratories. This did not include serotyping. The indication for testing samples varied over the time and were done due to clinician request, evidence of CSF pleocytosis, or clinical concerns about suspected meningitis. Recovery outcomes, including “full recovery,” were determined by the local clinical teams using existing data on the electronic clinical records.

Outcome ascertainment was based on hospital electronic health records. Outcome follow-up was based on clinically indicated care; this comprised specialist follow-up in a minority that was arranged due to clinical concerns, with a wider cohort where identification was based on subsequent health care contact resulting from re-presentation to a health care worker. Hospital records were monitored from the time of admission until the end of the study period, ranging from 1 to 11 years of longitudinal follow-up. Routine specialist follow-up is not recommended after viral meningitis. The study did not include direct access to primary care records, although correspondence from primary care clinicians, such as referral or clinic letters, was available within hospital records. Follow-up reflects UK practice within the NHS where primary care clinicians act as the principal point of ongoing surveillance and re-refer patients where clinically relevant concerns exist. Adverse outcomes at any time during the study period included the presence of seizures, motor sequelae, headache, academic impairment, hearing loss, speech delay, or other. This was defined as diagnosed per the treating clinician, which may include clinical diagnoses, examination, or routinely used clinical diagnostic tests. This included inpatient and outpatient assessment. The study had approval as a service evaluation by the Great Ormond Street Hospital Research and Development service (registration 3361).

Data were cleaned in Stata version 17. In handling missing data, denominators were the total where data were available for most outcomes. For severe outcomes (death and intensive care) and where detailed data were not available, the denominator was the whole population for that question; that is, we assumed that no response to intensive care admission meant that the patient was not admitted to intensive care and that no response to death meant that the patient did not die. This assumes that severe outcomes will have been identified. For comparison between groups, the χ^2^ test was used for categorical data and the Mann-Whitney *U* test for continuous data given the nonnormal distribution. Binary logistic regression was used to perform multivariable analysis of factors associated with full recovery. Candidate variables were identified by clinical relevance and used in univariate association by purposeful selection, as well as established confounders. More complex models including severity indicators and laboratory markers were explored, although these resulted in separation due to sparse outcome events. To avoid overfitting, the final model was restricted to age, sex, fever, seizures, baseline comorbidities, and critical care admission.

## RESULTS

A total of 243 patients with confirmed EV meningitis were included. The majority (200/243, 82.3%) were <3 months old at diagnosis. Males composed a higher proportion (137/243, 56.3%; Table 1). Baseline comorbidities at the time of diagnosis were present in 23% (56/243). Fever (195/243, 80.2%), difficulty feeding (70/243, 28.8%), and lethargy (43/243, 17.5%) were the most commonly described symptoms at presentation (Supplementary Appendix Table 1). There was a range of missingness, with high completion for age, sex, and acute clinical variables. Outcome data were available for 189 of 243 patients and long-term active specialist follow-up for 85 of 243.

**Table 1.** Clinical Characteristics and Outcomes of Children With Enterovirus Meningitis in the Full Cohort and Those With Tertiary Hospital Follow-up

			Those With Tertiary Hospital Follow-up (n & 85)				
	Full Cohort (n = 243)		Fully Recovered (n = 68)		Adverse Outcomes (n = 17)		
	No.	%	No.	%	No.	%	*P* Value^a^
Sex: male	137	56.4	42	61.8	6	35.3	.10
**Age, mo**							**<.01**
<3	200	82.3	61	89.7	7	41.2	
3–11	7	2.9	5	7.4	3	17.6	
12–59	16	6.6	1	1.5	5	29.4	
≥60	10	4.1	0	0.0	2	11.8	
**Clinical features**							
Fever	195	80.2	57	83.8	7	41.2	**<.01**
Irritability	36	14.8	12	17.6	1	5.9	.31
Difficulty feeding	70	28.8	23	33.8	17	100.0	**<.01**
Breathing difficulty	16	6.6	6	8.8	1	5.9	.76
Lethargy	43	17.7	12	17.6	1	5.9	.27
Seizures	15	6.2	2	2.9	7	41.2	**<.01**
Vomiting/diarrhea	23	9.5	7	10.3	1	5.9	.64
Any comorbidities	56	23.0	17	25.0	11	64.7	**<.01**
Ex-prematurity	19	7.8	7	10.3	3	17.6	.80
**ICU^b^**							
Admission	33	13.6	7	10.3	6	35.3	**.03**
Ventilated	30/33	90.9	5/7	71.4	6/6	100.0	**<.01**
Inotropes	4/33	12.1	0/7	0.0	1/6	16.7	**.04**
**Imaging findings^c^**							
Normal: USS brain	15/21	71.4	5/13	38.5	3/4	75.0	.51
Normal: MRI brain	2/20	10.0	2/7	28.6	0/8	0.0	.16
**Laboratory markers^d^**							
CSF WCC raised >20 × 10 mm^3^ L^−1^	67	27.6	22	32.4	5	29.4	.88

	Median	IQR	Median	IQR	Median	IQR	
**Maximum**							
CRP, mg/L	68	70	73.5	76	54	44	.22
WCC, ×10 mm^3^ L^−1^	10.1	4.5	10.1	6.4	14.85	4.7	.04

Data availability varied by variable. Percentages were calculated by the total number of participants in each cohort as the denominator, regardless of data availability for individual variables. Where data were missing, participants were retained in the denominator to reflect the real-world nature of follow-up. The number of participants with available data for each variable is shown in the numerator.

Abbreviations: CRP, C-reactive protein; CSF, cerebrospinal fluid; ICU, intensive care unit; MRI, magnetic resonance imaging; USS, ultrasound scan; WCC, white cell count.

^a^ *P* values were calculated with the χ^2^ test for categorical variables and the Mann-Whitney test for continuous variables. Bold indicates *P* < .05.

^b^ICU subanalyses for ventilation and inotrope use are restricted to those admitted to the ICU.

^c^Imaging results were available for 21 participants in the full cohort, 13 in the recovered group, and 4 in the adverse outcome group for cranial ultrasound and for 20, 7, and 8 participants for MRI, respectively.

^d^CSF white cell count was available for 243 participants in the full cohort, 68 in the recovered group, and 17 in the adverse outcome group.

### Diagnostic Findings

On admission, the median C-reactive protein was 68 mg/L (IQR, 70; reference range, <5 mg/L), and median white cells were 10.1 × 10^9^/L (IQR, 4.5; reference range, 4.0–11.0 × 10^9^/L). CSF white cells were >20 × 10^6^/L in 27.6% of participants (117/243) and >5 × 10^6^/L in 39.9% (97/243; reference range, 0–5 × 10^6^/L). CSF white cells had a range of 0.5 to 5920 × 10 mm^3^. One case had group B *Streptococcus* isolated in blood culture (maximum C-reactive protein, 35 mg/L; maximum white cell count, 15.3 ×10 mm^3^; CSF white cell count, 5 ×10 mm). All other sterile site cultures showed no significant growth. Of those who underwent neuroimaging during the acute episode, brain magnetic resonance imaging abnormalities were reported in 90% (18/20) of patients and abnormal brain ultrasound findings in 28.6% (6/21).

### Treatment and Hospital Course

Overall, 13.6% (33/243) of patients required admission to intensive care. Of these, the majority (30/33, 90.1%) required mechanical ventilation with a median duration of 3 days (IQR, 2). Four patients (4/33, 12.1%) required inotropes with a median duration of 2.5 days (IQR, 5).

Among patients without known comorbidities at baseline, the majority received no specific antiviral treatments, intravenous immunoglobulin, or steroids (160/187, 85.6%). Single-agent antiviral treatments were administered to 1 patient who received favipiravir for severe combined immune deficiency, 1 patient who received valganciclovir as concurrent treatment for cytomegalovirus, 1 patient who had dual therapy with acyclovir and brincidofovir for major histocompatibility complex 1 immune deficiency, and 1 patient who received pocapavir with no known comorbidities while in the intensive care unit (ICU; Supplementary Appendix Table 2). Six patients received steroids (median age, 20.5 months; IQR, 26). Three patients received intravenous immunoglobulins (median age, 5 months; IQR, 18): 1 patient, 2 doses acutely; 1 patient, 24 doses for underlying condition; and 1 patient, unknown doses given acutely.

### Outcomes

The median recorded follow-up length was 16 months (IQR, 30) among those with documented routine specialist outpatient follow-up. Of 243 patients, 85 (35.0%) had active tertiary hospital routine follow-up documented, and the rest of the cohort had outcomes ascertained through routine health records, capturing re-presentation, primary care correspondence, or readmissions. Adverse sequelae based on passive follow-up (re-presentation or readmission) were reported in a minority of the whole cohort (17/189, 8.9%) and in 1 patient (1/126, 0.8%) <3 months old who had no comorbidities and did not require ICU admission (Supplementary Appendix Table 3). Among patients with documented tertiary follow-up, the proportion was higher, consistent with selective follow-up of patients with greater clinical concern (17/85, 20%). Of those with adverse sequelae, 6 of 17 (35.3%) were admitted to the ICU, and 11 of 17 (64.7%) had comorbidities at baseline. A further 2 of 17 (11.7%) had additional medical conditions identified at a later time point, such as inner ear pathology and a temporal lobe intracranial tumor. A higher proportion of comorbidities were observed in those without documented full recovery (13/17, 76.5%). The following variables were associated with a lower likelihood of a full recovery: older age, absence of fever, feeding difficulty, seizures, presence of comorbidities at baseline, and admission to ICU (Table 1). Of 160 patients, 153 (95.6%) aged <3 months had full recovery, as compared with 5 of 7 (71.4%) aged >5 years.

On multivariate analysis, adverse outcomes were associated with the absence of fever at presentation (odds ratio [OR], 4.65; 95% CI, 1.03–20.83), the presence of seizures (OR, 7.40; 95% CI, 1.05–51.96), and the presence of comorbidities (OR, 5.27; 95% CI, 1.18–23.47; see supplementary appendix).

In those with adverse sequelae, the most common developmental impairment was persistent motor dysfunction including hypotonia, observed in 7 patients (Supplementary Appendix Table 4). Of the 7 patients with motor impairment, 5 had comorbidities at baseline that may have contributed (eg, Haddad central hypotonia), and 1 had previous febrile seizures associated with status epilepticus (Supplementary Appendix Table 5). We did not have any formal diagnoses of acute flaccid myelitis, although there were infants with transient hypotonia. Five had academic impairment (eg, social-emotional and speech related); of these, 3 had significant comorbidities at the time of presentation that may have contributed to baseline impairment, and 1 had suspected coinfection with varicella at the time of illness that may have contributed. Two patients had hearing loss at follow-up, although these may be explained by other factors, as 1 had congenital cytomegalovirus and the other conductive hearing loss requiring ear-nose-throat surgery. One patient died, who was 2 years old with complications of underlying major histocompatibility complex class II immunodeficiency.

## DISCUSSION

We describe long-term outcomes of EV meningitis in a large cohort of children across a 10-year surveillance period. The findings are broadly reassuring with the majority of children recovering without complications. Of the 17 children without full recovery, 13 had another condition or comorbidity (eg, prematurity, neurologic, or known immune deficiency) that may have contributed to their sequelae.

Our study supports the existing literature and demonstrates a reassuring lack of sequalae following EV meningitis: a systematic review had found minimal evidence of significant sequelae following viral meningitis among mostly children without comorbidities, although there were significant heterogeneities and biases in the data presented [4, 5]. This contrasts with data from Huang et al [6], who reported severe outcomes in those affected by EV71 meningitis, which may be due to higher neurotropism and exaggerated cytokine-induced inflammatory response in this specific EV type [7]. Receptors unique to EV71 such as SCARB2 (scavenger receptor class B member 2) that mediate virus binding to neuronal cells are thought to play a central role in the more severe neurologic outcomes of EV71 infections as compared with non-EV71 infections [8]. In 1 case series, 9 of 16 (56%) patients had long-term poliomyelitis-like syndrome following EV71 infection [9]. While severe EV71 outbreaks have been commonly reported in the Asia-Pacific region, there have also been outbreaks of severe neurologic disease associated with EV71 in European countries [10, 11]. Specific genotyping was not conducted: EV71 is infrequently circulating in this cohort and not anticipated to represent a significant burden, while Coxsackie virus and echovirus are more commonly associated with European outbreaks [10, 11].

Our study describes a large multisite case series to examine long-term outcomes in children with EV meningitis, who were enrolled over a decade across a diverse capital city, with children from multiple ethnicities, ages, and socioeconomic groups. Robust database linkage between clinical and laboratory records helped optimize case ascertainment of eligible cases. We had a strict case definition that supports the validity of our findings. However, limitations include retrospective data collection, which may introduce confounding bias, and enrollment in 4 tertiary London centers, which could introduce selection bias. Most patients were infants, which makes it difficult to generalize to other age groups. Although we were not powered to analyze this, testing for viral meningitis likely increased at a national level among infants during the study period, which may reduce the generalizability of our results. Infants aged <3 months with fever are often evaluated differently, including routine lumbar punctures in UK clinical practice, which may partially explain the preponderance of infants in this cohort. We did not have confirmed cases of acute flaccid myelitis and therefore cannot make any conclusions about this syndrome in this study. Expansion of multiplex platforms in standard panels for febrile infants may have led to increased identification in younger groups [12]. There are limited follow-up data and we could confirm outcomes beyond hospital discharge in only 85 (35.0%) of 243 cases; the remainder were passive data and we did not determine whether specific follow-up tests were performed (eg, hearing test) when initial findings were normal. This is due to the lack of routine prospective follow-up being recommended in this patient group. We would, however, have had data if patients re-presented to the same hospital with severe outcomes. This is a justifiable approach in a typical health care setting, particularly as we would expect general practitioners to commonly refer back to specialists in the case of any postdischarge outcomes requiring medical attention. We did not collect genotyping data or aggregate data based on immune status. Our data are limited by a reliance on clinically obtained administrative data, a lack of uniform testing across hospitals based on clinical discretion, and missing data.

Our study should provide some reassurance that long-term adverse outcomes are unlikely in the majority of children with EV meningitis, especially in the absence of baseline comorbidities or ICU admission, and it suggests that there is no obvious need for follow-up beyond hospital discharge in cases of EV meningitis in this specific cohort. However, we found that a minority of children with baseline comorbidities and those requiring ICU admission had neurodevelopmental impairment following EV meningitis. These children would benefit from longer-term pediatric follow-up to monitor neurodevelopmental outcomes. Our data highlight the need for further robust prospective follow-up studies of children with EV meningitis to identify which are most at risk of developing long-term neurodevelopmental impairment and to define any association with genotype and thus potentially inform development of future treatment and prevention trials.

## Supplementary Material

ofag112_Supplementary_Data

## Supplementary Data

Supplementary materials are available at *Open Forum Infectious Diseases* online. Consisting of data provided by the authors to benefit the reader, the posted materials are not copyedited and are the sole responsibility of the authors, so questions or comments should be addressed to the corresponding author.

## References

[1] Kohil A, Jemmieh S, Smatti MK, et al Viral meningitis: an overview. Arch Virol 2021; 166:335–45. 33392820 10.1007/s00705-020-04891-1 PMC7779091

[2] Martin N, Defres S, Willis L, et al Paediatric meningitis in the conjugate vaccine era and a novel clinical decision model to predict bacterial aetiology. J Infect 2024; 88:106145. 38552719 10.1016/j.jinf.2024.106145

[3] Kadambari S, Braccio S, Ribeiro S, et al Enterovirus and parechovirus meningitis in infants younger than 90 days old in the UK and republic of Ireland: a British Paediatric Surveillance Unit study. Arch Dis Child 2019; 104:552–7. 30530486 10.1136/archdischild-2018-315643

[4] Hudson JA, Broad J, Martin NG, et al Outcomes beyond hospital discharge in infants and children with viral meningitis: a systematic review. Rev Med Virol 2020; 30:e2083. 31524309 10.1002/rmv.2083

[5] Verboon-Maciolek M, Krediet T, Van Loon A, et al Epidemiological survey of neonatal non-polio enterovirus infection in the Netherlands. J Med Virol 2002; 66:241–5. 11782934 10.1002/jmv.2136

[6] Huang CC, Liu CC, Chang YC, et al Neurologic complications in children with enterovirus 71 infection. N Engl J Med 1999; 341:936–42. 10498488 10.1056/NEJM199909233411302

[7] Lai RH, Chow YH, Chung NH, et al Neurotropic EV71 causes encephalitis by engaging intracellular TLR9 to elicit neurotoxic IL12-p40-iNOS signaling. Cell Death Dis 2022; 13:328. 35399111 10.1038/s41419-022-04771-3 PMC8995170

[8] Yamayoshi S, Yamashita Y, Li J, et al Scavenger receptor B2 is a cellular receptor for enterovirus 71. Nat Med 2009; 15:798–801. 19543282 10.1038/nm.1992

[9] Chang LY, Huang LM, Gau SSF, et al Neurodevelopment and cognition in children after enterovirus 71 infection. N Engl J Med 2007; 356:1226–34. 17377160 10.1056/NEJMoa065954

[10] González-Sanz R, Casas-Alba D, Launes C, et al Molecular epidemiology of an enterovirus A71 outbreak associated with severe neurological disease, Spain, 2016. Euro Surveill 2019; 24:1800089. 30782267 10.2807/1560-7917.ES.2019.24.7.1800089 PMC6381658

[11] Puenpa J, Wanlapakorn N, Vongpunsawad S, et al The history of enterovirus A71 outbreaks and molecular epidemiology in the Asia-Pacific region. J Biomed Sci 2019; 26:1–11. 31627753 10.1186/s12929-019-0573-2 PMC6798416

[12] Kadambari S, Abdullahi F, Celma C, Ladhani S. Epidemiological trends in viral meningitis in England: prospective national surveillance, 2013–2023. J Infect 2024; 89:106223. 38986749 10.1016/j.jinf.2024.106223

