# Supplementary material for: Outcomes in Children With Enterovirus Meningitis in London, England: A Retrospective Multicenter Cohort Study, 2013–2023
Source: Open Forum Infect Dis. 2026 Mar 9;13(3):ofag112. doi: 10.1093/ofid/ofag112 (PMC13001589; doi:10.1093/ofid/ofag112)
Supplement: ofag112_Supplementary_Data [file ofag112_supplementary_data.docx]

**Appendix table 1: Treatments received amongst 36 with defined treatment (n=36)**

| **Therapy** | **Number** |
| --- | --- |
| **Aciclovir** | **36** |
| **Favipiravir** | **1** |
| **Valganciclovir** | **1** |
| **Aciclovir with Brincidofovir** | **1** |
| **Pocapapavir** | **1** |
| **Received IVIG** | **3** |
| **Received steroids** | **6** |
| **--For a known prior condition** | **4** |
| **--Without a known prior condition** | **2** |

**Appendix table 2: Adverse sequelae by patient group (n=189)**

| **Patient group** | **Adverse sequelae n/N (%)** |
| --- | --- |
| **All patients with data** | 17/189 (8.9%) |
| **Non-primary immunodeficiency** | 15/187 (8.02%) |
| **Non-ICU patients** | 11/175 (6.3%) |
| **Infants <3 months** | 7/160 (4.38%) |
| **No previous comorbidities** | 5/141 (3.55%) |
| **Previously Healthy Infants, <3 Months, Non-ICU** | 3/126 (2.38%) |
| **Previously Healthy Infants, <3 Months, Non-ICU, No Illness During Follow-Up** | 1/126 (0.79%) |

**Appendix table 3: Adverse sequelae amongst 17 patients without full recovery (n=17)**

| **Outcome** | **Number/Total** | **Percent (%)** |
| --- | --- | --- |
| Academic impairment | 5/17 | 29.4% |
| Motor impairment | 7/17 | 41.2% |
| Seizures persist | 4/17 | 23.5% |
| Speech deficit | 4/17 | 23.5% |

**Appendix table 4: Co-morbidities amongst patients with adverse sequalae (n=17). Where patients have more than one co-morbidity this is recorded, therefore numbers do not sum 100%.**

| **Co-morbidities (n=17)** | **N** | **Proportion (%)** |
| --- | --- | --- |
| **Prematurity** | 2 | 11.8% |
| **Respiratory disease** | 2 | 11.8% |
| **Neurological disease** | 2 | 11.8% |
| **Malignancy** | 1 | 5.9% |
| **Metabolic disease** | 1 | 5.9% |
| **Inborn error of immunity** | 2 | 11.8% |
| **Congenital infection** | 1 | 5.9% |
| **Any co-morbidity at baseline** | 11 | 64.7% |

**Appendix table 5: Multivariate logistic regression of factors associated with full recovery for those with data on outcomes (n=189)**

|  | **Odds ratio** | **Lower 95% CI** | **Upper 95% CI** | **P value** |  |
| --- | --- | --- | --- | --- | --- |
| **Age (months)** | 0.99 | 0.96 | 1.01 | 0.307 |  |
| **Female sex** | 0.47 | 0.12 | 1.85 | 0.283 |  |
| **Absence of fever** | 4.65 | 1.03 | 20.8 | **0.045** |  |
| **Seizures** | 7.40 | 1.05 | 51.96 | **0.044** |  |
| **Comorbidities** | 5.27 | 1.18 | 23.47 | **0.029** |  |
| **ICU admission** | 1.05 | 0.13 | 8.87 | 0.963 |  |

Odds ratios presented are from a multivariate logistic regression with full recovery as the binary outcome. Age was used as a continuous variable in months. Other variables were treated as binary categorical variables. Odds ratios greater than 1 indicate higher odds of adverse outcomes, and lower than 1 indicate lower odds of full recovery. Known predictors were included, except covariates that led to complete separation (including white cells and feeding difficulties).
